# Effect of probiotic *Pediococcus acidilactici* FT28 on growth performance, nutrient digestibility, health status, meat quality, and intestinal morphology in growing pigs

**DOI:** 10.14202/vetworld.2018.1669-1676

**Published:** 2018-12-14

**Authors:** Mamata Joysowal, B. N. Saikia, Runjun Dowarah, S. Tamuly, D. Kalita, K. B. Dev Choudhury

**Affiliations:** 1 Department of Animal Nutrition, College of Veterinary Science, Assam Agricultural University, Khanapara, Guwahati, Assam, India; 2 Department of Veterinary Biochemistry, College of Veterinary Science, Assam Agricultural University, Khanapara, Guwahati, Assam, India; 3 ICAR-AICRP on Pigs, Faculty of Veterinary Science, Assam Agricultural University, Khanapara, Guwahati, Assam, India; 4 Department of Veterinary Anatomy and Histology, College of Veterinary Science, Assam Agricultural University, Khanapara, Guwahati, Assam, India

**Keywords:** growth performance, intestinal morphology, meat quality, *Pediococcus acidilactici*, pig

## Abstract

**Aim:**

The experiment was conducted to evaluate the effect of swine-origin probiotic *Pediococcus acidilactici* FT28 on growth, nutrient utilization, health status, meat quality and intestinal morphology in growing female pigs.

**Materials and Methods:**

Pigs (n=27) were distributed into three groups (3 replicates of 3 each) and supplemented with basal diet either without probiotics (C) or with a probiotic of dairy-based (*Lactobacillus acidophilus* NCDC-15; *TLact*) or swine based (*P. acidilactici* FT28; *TPedic*). The probiotics were fed as fermented feed at 200 g/pig/day. At the end of the trial, six pigs from each group were selected for metabolism trial and then sacrificed to determine meat quality and intestinal morphology.

**Results:**

Supplementation of both probiotics improved growth performance, whereas feed intake, digestibility of CP and N retention were better (p<0.05) in *P. acidilactici* FT28-fed group. However , the digestibility of dry matter (DM), organic matter (OM), ether extracts (EE), crude fiber and nitrogen free extract did not show any significant effect on probiotic supplementation. The serum A: G ratio, triglyceride, and cholesterol level were also improved (p<0.05) in *TPedic* group compared to other treatment groups. Both probiotic supplementations showed lower (p<0.05) serum glucose level with similar protein and albumin value, which indicated good utilization of feed as well as health status of growing pigs. Dressing percentage, vital organ weight, and EE of loin meat were higher (p<0.05) in probiotic-supplemented groups compared to control. However, *P. acidilactici* FT28-fed animals showed higher (p<0.05) CP and total ash percentage of meat without affecting pH, water holding capacity, and extract release volume of loin muscle. The villi height and crypt depth were better in both supplemented groups compared to control.

**Conclusion:**

Results of the present study revealed that *P. acidilactici* FT28 could serve as better probiotic source in swine production for the better utilization of CP and N-retention in meat with improved health status and intestinal morphology.

## Introduction

Due to ban on the use of antimicrobial growth promoters, the application of probiotics had gained significant attention in developing suitable alternatives of antibiotics in the swine industry [1-3]. Probiotics are live microbial feed supplement, which beneficially affects the host animal by improving its microbial balance [4]. The lactic acid bacteria (LAB) either in pure or mixed cultures are commonly used as probiotics. Dietary supplementation of LAB improved the growth performance, nutrient digestibility, blood biochemical and immune profile, intestinal microbial balance, and intestinal morphology in growing pigs [5-8]. However, the effect of LAB as probiotics in practice is highly inconsistent due to variation in composition of diet, strain-specificity, doses, age of animal, and environmental effect [8-10].

In this context, recent studies reveal that feeding of probiotics of host origin (*Lactobacillus johnsonii*, *Lactobacillus mucosae*, *Lactobacillus* *plantarum*, and *Pediococcus acidilactici*) improved growth, feed conversion ratio (FCR), nutrient digestibility, gut health with decreased *Escherichia coli*, and increased *Lactobacilli* shedding feces of weaned piglets [9,11] and grower-finisher pigs [8,11]. The better response of host origin probiotic might be obtained due to better adhesion and colonization in the intestine with species-specific strains [9,12].

Therefore, the present study was carried out to evaluate the comparative effcacy of dairy (*L. acidophilus* NCDC15) and swine (*P. acidilactici* FT28)-based probiotic for improving growth, nutrient digestibility, health status, meat quality, and intestinal health in growing crossbred pigs.

## Materials and Methods

### Ethical approval

The present study was conducted in the ICAR-MSP Swine Farm, College of Veterinary Science, Assam Agricultural University, Guwahati, India. The animal experimental protocol was approved by the Institutional Animal Ethics Committee (IAEC) with approval No. 770/ac/CPCSEA/FVSc/AAU/IAEC/16-17/430 and carried out as per the guidelines of the Committee for the Purpose of Control and Supervision of Experiments in Animals (CPCSEA), Ministry of Environment, Forests and Climate Change, Government of India.

### Source of probiotics

A culture of *L. acidophilus* NCDC 15 (conventional dairy origin) was obtained from culture collection laboratory of NDRI, Karnal. *P. acidilactici* FT 28 (swine origin) was procured from IVRI, Izatnagar, which was previously isolated from weaned piglet feces and identified by 16S RNA gene sequence (Accession No. KU837246) with *in vitro* probiotic properties such as tolerance to pH and bile salt, cell surface hydrophobicity, and antagonism to bacterial pathogens [13].

### Preparation of probiotic product

The probiotics product was prepared as per the procedure described by Agarwal *et al*. [14]. 1 kg ground maize was mixed with 1 L distilled water and inoculated with 24 h old culture (100 ml) of either *L. acidophilus* NCDC15 or *P. acidilactici* FT28 and kept for 24 h at 37°C for fermentation. Next day, the same fermented maize (20% [w/w]) was used as inoculums for the preparation of fermented feed for coming days and such process was continued for 10 days. After 10 days, a new vial of culture was used to prepare fermented feed. The colony-forming unit (CFU/g) in fermented feed was counted at every alternate day to check the viability of bacterial cells and was maintained at the level of 1-2×10^9^.

### Experimental design, animals, and housing

A total of 27 female crossbred HD K-75 (Hampshire x local) grower pigs with an average initial weight of 18.33±0.93 kg were procured from AICRP on Pigs, Assam Agricultural University, Guwahati, Assam, India. The piglets were assigned into three dietary groups (3 replicates of 3 each) in a randomized block design. Dietary treatments included control fed basal diet, *TLacto* (basal diet + *L. acidophilus* NCDC15; conventional dairy-origin probiotic), and *TPedic* (basal diet + *P. acidilactici* FT28; swine-origin probiotic). The piglets were housed on cemented floor pens provided with separate feeder and water tough facilities. Before housing of the piglets, the floor pens were thoroughly disinfected using fumigants and flame gun. Mortality, if any, was recorded.

### Experimental diet and feeding regimen

The basal diet was formulated as per the ICAR [15] recommendation (Table-1). The probiotic product (2.0×10^9^ CFU/g) was mixed in the basal diet and offered at 200 g/day/pig, which was fed in the morning (08.30 h) by subtracting the equal amount of maize from basal diet. Fresh feed and clean water were offered *ad libitum* throughout the experimental period.

**Table-1:** Ingredient and chemical composition (% DM basis) of experimental basal diet for grower pigs.

Attributes	% DM basis
Ingredient composition	
Maize	55
Wheat bran	20
De-oiled groundnut cake	10
Soybean meal	13
Mineral mixture*	1.5
Salt	0.5
Chemical composition	
OM	91.90
CP	15.50
CF	5.65
EE	4.00
NFE	66.75
Total ash	8.10
Calcium	2.70
Phosphorus	0.79

* Each 1 kg contain: Vitamin A 2,000,000 IU, Vitamin D_3_ 400,000 IU, Vitamin B_2_ 0.8 g, Vitamin E 0.3 g, Vitamin K 0.4 g, Vitamin B_12_ 2.4 mg, calcium pantothenate 0.1 mg, niacin 4 g, choline chloride 60 g, calcium 0.28 g, manganese 11 g, iodine 0.4 g, iron 3 g, zinc 6 g, copper 0.8 g, cobalt 0.18 g, phosphorus 80 g, DM=Dry matter OM=Organic matter, EE=Ether extract, CF=Crude fiber, NFE=Nitrogen-free extract, CP=Crude protein

### Growth performance

Daily feed offered and residue left was weighed and recorded to monitor daily feed intake. Pigs were weighed individually at fortnight intervals during the entire experimental period of 90 days to calculate average final body weight (FBW), average daily gain (ADG), and FCR.

### Nutrient digestibility

A metabolism trial was conducted at the end of the experimental feeding for 5 days (2 day adaptation + 3 day collection) to assess the digestibility and retention of nutrients. Six animals were selected from each group with comparable weights and then transferred to individual metabolism cages. The feed offered and residue left were collected daily and pooled over 3 days, and subsamples were further used for the analysis. The feces from each animal were collected manually immediately after defecation and pooled for 24 h and weighed daily at 8.30 AM. The fecal subsamples were collected daily and dried in hot air oven at 60±5°C for 24-48 h. Another 10% of feces were stored in 1:4 H_2_SO_4_ (10% v/w) for the estimation of nitrogen and crude protein (CP). The total urine voided within 24 h was collected in dilute H_2_SO_4_ (1:4) and a suitable aliquot (1/10^th^) was kept in reagent bottle for nitrogen estimation. The feed, residue, and feces samples were ground and analyzed for proximate principle [16].

### Blood biochemical profile

Blood samples were collected from cranial vena cava in the morning (before watering and feeding) into a vacutainer tube from all the pigs at 30, 60, and 90 days of feeding trial. Serum was separated from whole blood by centrifugation at 3000× g for 30 min. The metabolites such as glucose, total protein, albumin, globulin, A/G ratio, cholesterol, and triglycerides were determined colorimetrically using commercial diagnostic kits (Coral Clinical System, Goa, India) by spectrophotometer model UV-2601, Labomed, USA.

### Carcass traits

The selected animals were fasted for 12 h and transported to an experimental abattoir of the institute, where the pre-slaughter weight (PSW) was recorded. The pigs were slaughtered by electric stunning (250 mA), and complete drainage of blood was done by heart puncturing. The head, hair, and viscera were removed from carcass and then made into two vertical separate halves. After evisceration, dressed carcass weight was recorded without head and shanks and expressed as a percentage of PSW. The visceral organs (liver, heart, spleen, and kidney) were weighed and expressed as a percentage of PSW [17].

### Physicochemical properties of meat

For evaluation of physicochemical properties, the ham muscle was collected from the dressed carcass after 30 min of postmortem and frozen at −20°C before subsequent analysis.

### pH measurement

About 10 g of fresh meat was minced and mixed with 90 ml distilled water and blended in tissue homogenizer. The pH of the suspension was recorded using a digital pH meter. The probe of pH meter was calibrated using two buffers (pH 4.0 and 7.0), and each measurement was repeated 3 times.

### Water holding capacity (WHC)

WHC was measured by centrifugation method [18] using ice cold 0.6M NaCl. About 100 g of meat was used to make a slurry and centrifuged at 1500× g for 10 min. The supernatant volume (V) was collected and expressed as the amount of added solution (ml) retained by 100 g of meat.

### Extract release volume (ERV)

About 25 g ground meat sample was homogenized in high speed (15,000 rpm) in Waring Blender for 2 min with 100 ml of distilled water. The homogenate was then filtrated through Whatman No.1 and collected for 15 min. Average of two volumes is considered as ERV.

### Chemical composition of meat

The moisture content of the collected sample was determined by oven drying, fat by Soxhlet extraction with petroleum ether (40-60°C b.p.), and CP by Kjeldahl nitrogen estimation [16].

### Intestinal morphology

At the end of the experiment, six animals from each group were slaughtered to evaluate the carcass parameters and intestinal morphology. The entire intestinal tracts were removed; the jejunum was collected from each animal and promptly fixed in 10% neutral buffered formalin. The specimens were then dehydrated in graded alcohols, cleared with xylene, and embedded in paraffin, and serial microtome sections (6 µm thick) were stained with hematoxylin and eosin stain which was examined at 10× and 100× magnification under a light microscope to assess villus height and crypt depth [8].

### Statistical analysis

The statistical analysis of the experimental data was carried out using Statistical Package for the Social Science (version 17.0 for Windows; SPSS, Chicago, III., U.S.A.). The one- and two-way analysis of variance (ANOVA) was used to compare the means at 5% level of significance according to Duncan’s multiple range test [19].

## Results

### Growth performance

Supplementation of both the probiotics improved (p<0.05) FBW, ADG (g/d), and FCR in crossbred grower pigs (Table-2). The average feed intake (g/d) was higher (p<0.05) in *TPedic* group compared to control, where *TLacto* group showed comparable values with other groups.

**Table-2:** Effect of probiotics on growth performance and nutrient digestibility in grower pigs.

Attribute	Treatment*			p-value

	C	*TLacto*	*TPedic*	
Growth performance				
Initial BW (kg)	18.35±1.90	18.41±1.79	18.24±1.61	0.998
FBW (kg)	50.63^a^±1.96	56.06^b^±2.14	56.89^b^±1.01	0.043
ADG (g/day)	358.6^a^±17.6	418.3^b^±19.3	429.4^b^±19.2	0.030
FI (g/d)	684.0^a^±2.11	727.5^b^±1.94	762.4^c^±1.33	<0.001
FCR	4.08^b^±0.09	3.52^a^±0.014	3.49^a^±0.066	0.014
Performance at metabolism trial				
BW/kg^0.75^	19.17±0.11	20.81±0.10	20.96±0.10	0.101
DMI/kg^0.75^	80.36±0.88	74.35±0.91	75.61±4.38	0.308
Apparent nutrient digestibility (%)				
Dry matter	74.66±0.95	74.26±1.57	76.38±0.90	0.449
OM	75.48±0.71	76.32±0.32	77.61±0.96	0.187
EE	77.83±0.39	80.16±1.40	81.15±1.58	0.236
CF	23.56±2.60	24.71±5.29	33.93±2.47	0.174
NFE	82.25±0.31	82.31±0.16	82.79±0.38	0.167
CP	66.26^a^±1.06	69.91^ab^±1.58	72.94^b^±1.25	0.032
N retention	62.71^a^±1.33	65.59^ab^±1.14	67.97^b^±0.94	0.049

* No probiotics (C), *Lactobacillus acidophilus* (*TLact*), *Pediococcus acidilactici* FT28 (*TPedic*),

ab Means bearing different superscripts in a column differ significantly (p<0.05), OM=Organic matter, EE=Ether extract, CF=Crude fiber, NFE=Nitrogen-free extract, FCR=Feed conversion ratio, ADG=Average daily gain, FBW=Final body weight, CP=Crude protein

### Nutrient digestibility

During metabolism trial, the average metabolic body weight and dry matter (DM) intake (kg/kg metabolic body weight) were similar between the treatment groups (Table-2). The total tract apparent digestibility of DM, organic matter (OM), EE, CF, and nitrogen free extract (NFE) was not differed (p>0.05) among the dietary groups. However, the digestibility of CP and nitrogen retention was superior (p<0.05) in pig fed *P. acidilactici* FT28 compared to control. *TLacto* group (*L. acidophilus* NCDC15) showed comparable results with *TPedic* and control (C) groups.

### Blood biochemical profile

Serum concentration of glucose was decreased (p<0.001) in probiotic-supplemented groups (*TLacto* and *TPedic*) compared to control (Table-3). The significant effect was also observed due to the effect of period and interaction of treatment x period. However, the concentration of serum total protein and albumin was not differed among the treatment groups. The serum globulin and albumin-to-globulin (A: G) ratio were significantly higher (p<0.05) in *TPedic* groups by supplementing *P. acidilactici* FT28 in grower pigs compared to other treatment groups. Serum concentration of triglycerides and cholesterol was lower (p<0.05) in *TPedic* group animals in comparison to control and *TLacto* groups. Cholesterol concentration in blood serum was differed significantly (p<0.05) among the treated animals due to the interaction of treatment x period.

**Table-3:** Effect of probiotics on blood biochemical profile in grower pigs.

Attributes	Period			Mean±SE	Significance		
	
	D-0	D-45	D-90		T	P	T*P
Glucose (mg/dL)							
C	109.3±3.25	110.3±0.65	112.3±0.65	110.6^b^±1.74	<0.001	<0.001	<0.001
*TLacto*	109.8±4.11	87.50±0.65	86.50±0.65	94.58^a^±3.48			
*TPedic*	111.8±3.59	92.50±3.25	91.50±1.96	95.58^a^±3.02			
Total protein (g/dL)							
C	10.23±6.46	10.79±7.59	8.95±6.59	9.99±0.57	0.115	0.800	0.170
*TLacto*	8.24±6.23	8.89±7.26	9.05±7.59	8.73±0.30			
*TPedic*	10.75±7.09	8.65±7.78	10.00±8.89	9.80±0.45			
Albumin (mg/dL)							
C	50.30±13.3	35.10±21.6	26.50±4.27	43.96±6.40	0.128	0.148	0.041
*TLacto*	27.57±5.71	33.65±17.7	29.70±16.5	30.38±2.95			
*TPedic*	54.28±14.9	22.75±13.3	36.50±21.4	37.84±5.65			
Globulin (mg/dL)							
C	52.00±0.41	52.75±0.75	63.00±0.71	55.92^a^±1.54	0.002	<0.001	0.002
*TLacto*	54.83±2.40	55.25±2.89	60.80±0.52	56.96^a^±1.30			
*TPedic*	53.25±0.95	63.50±1.19	63.50±1.29	60.83^b^±1.58			
Albumin:globulin ratio							
C	0.96±0.22	1.05±0.21	0.42±0.11	0.31^a^±0.130	0.040	0.036	0.024
*TLacto*	0.51±0.13	0.61±0.10	0.49±0.06	0.54^ab^±0.07			
*TPedic*	1.03±0.25	0.36±0.05	0.58±0.09	0.67^b^±0.12			
Triglycerides (mg/dL)							
C	55.25±6.42	70.25±4.05	72.75±3.97	66.08^b^±2.57	<0.001	0.208	0.484
*TLacto*	50.25±12.0	74.25±8.28	77.00±0.02	67.17^b^±3.47			
*TPedic*	32.75±5.28	71.00±6.68	70.25±4.87	58.00^a^±5.28			
Cholesterol (mg/dL)							
C	40.50±2.50	54.50±8.68	56.50±8.68	50.50^b^±4.35	0.011	0.807	0.006
*TLacto*	48.00±1.00	36.75±0.85	35.75±0.85	40.17^b^±1.74			
*TPedic*	45.75±0.95	37.00±1.68	36.00±1.68	39.58^a^±1.53			

* No probiotics (C), *Lactobacillus acidophilus* (*TLact*), *Pediococcus acidilactici* FT28 (*TPedic*),

ab Means bearing different superscripts in a column differ significantly (p<0.05)

### Carcass parameters

Grower pigs fed basal diet supplementation with probiotics (*TLacto* and *TPedic* groups) did not show any variation (p>0.05) on PSW when slaughtered at the end of the experimental feeding (Table-4). However, extra-cellular water (ECW), dressing percentage, and vital organ weight (% of ECW) were higher in both probiotic-supplemented groups compared to control. There was no significant variation observed among the probiotic-fed groups.

**Table-4:** Effect of probiotics on carcass characteristics, physicochemical parameters of meat, and intestinal morphology in grower pigs.

Attributes	Treatment*			p-value

	C	*TLacto*	*TPedic*	
PSW (kg)	68.50±0.50	71.0±1.00	72.00±2.00	0.299
ECW (Kg)	48.50^a^±0.50	55.50^b^±1.50	57.00^b^±2.00	0.050
Dressing percentage	70.81^a^±1.24	78.15^b^±1.01	78.86^b^±0.86	0.021
Vital organ weight (% of ECW)	8.40a±0.04	15.04^b^±0.04	14.23^b^±0.85	0.004
Physicochemical parameters				
pH	4.93±0.11	5.06±0.03	5.05±0.02	0.421
WHC (cm^2^)	6.14±0.62	4.24±0.06	3.95±0.35	0.059
ERV (ml)	68.00±14.00	56.00±6.00	52.50±7.50	0.568
DM (%)	24.51±0.03	28.28±2.34	25.08±0.08	0.252
CP (%)	19.96^a^±0.16	20.95^ab^±0.10	21.10^b^±0.35	0.031
EE (%)	2.65^b^±0.05	2.43^a^±0.03	2.43^a^±0.08	0.040
Total ash (%)	1.29^a^±0.01	1.37^b^±0.02	1.59^c^±0.01	0.001
Intestinal morphology				
Villi height (μm)	629.9^a^±22.97	680.0^b^±14.14	700.3^b^±2.57	0.027
Crypt depth (μm)	69.43^a^±2.56	71.39^b^±3.51	123.70^b^±8.52	0.024

* No probiotics (C), *Lactobacillus acidophilus* (*TLact*), *Pediococcus acidilactici* FT28 (*TPedic*),

ab Means bearing different superscripts in a column differ significantly (p<0.05), PSW=Pre-slaughter weight, WHC=Water holding capacity, ERV=Extract release volume, DM=Dry matter, CP=Crude protein, ECW=Extra-cellular water, EE=Ether extract

### Physicochemical properties of meat

The pH, WHC, and ERV in loin muscle were not shown any significant differences between the treatment groups (Table-4). However, the CP and total ash were greater (p<0.05) in *TPedic* group compared to control and *TPedic* groups. There was a significant variation observed between *P. acidilactici* FT28 and *L. acidophilus* NCDC15-supplemented animals, where earlier had higher CP and total ash content in the meat. EE content was decreased (p<0.05) in both the probiotic-fed groups without showing any variation on DM content in loin muscle.

### Intestinal morphology

**Figure-1:**
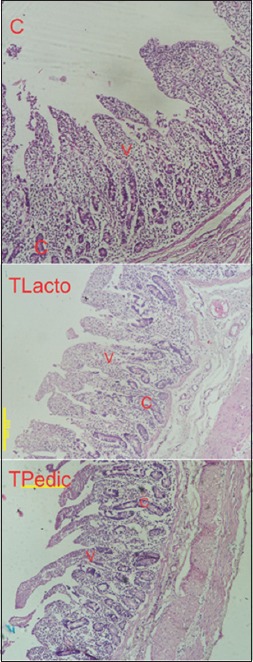
Change in intestinal morphology of grower pigs by feeding probiotics (hematoxylin/eosin stain 100×). The duodenum structure is regularly organized in villi (V) and crypt (C).

The histopathology evaluation of the duodenum showed that the villi height (µm) and crypt depth (µm) were improved (p<0.05) in probiotic-supplemented groups (dairy origin: *TLacto* and swine origin: *TPedic*) compared to control (Table-4 and Figure-1).

## Discussion

In the present study, gain in weight and daily weight gain in growing pigs were improved by the addition of *L. acidophilus* and *P. acidilactici* FT28 in the diet. A positive effect was also observed on FCR in both the probiotic (dairy and swine origin)-fed groups (Table-2). In the same line, Dowarah *et al*. [7] also observed higher net weight gain, ADG, and daily DM intake in early weaned grower-finisher pigs by supplementing *L. acidophilus* and *P. acidilactici*. The earlier several studies also reported higher gain in body weight and feed intake due to feeding of probiotic in growing pigs [20-22].

As a natural inhabitant, LAB present in gastrointestinal tract produces metabolites such as lactic acid and digestive enzymes, stimulate gastrointestinal peristalsis, and promote apparent nutrient digestibility [23]. In our experiment, apparent digestibility of CP and nitrogen retention were higher in *P. acidilactic*i FT28-fed group compared to control, which were comparable with the results of *L. acidophilu*s NCDC15-fed group (Table-2). However, apparent digestibility of DM, OM, CF, EE, and NFE not differed among the treatment groups (Table-2). Similarly, Yu *et al*. [24] and Datt *et al*. [21] observed better CP digestibility in growing pigs when a mixture of probiotics strain incorporated in the basal diet. Balasubhramaniam *et al*. [25] observed significant effects on nitrogen retention by supplementing *Bacillus*-based probiotics in growing-finishing pigs. In contrast to our study, higher nutrient digestibility was observed in pigs fed different probiotics (0.1% and 0.2%) and *L*. *acidophilus*, respectively [1,26].

Serum biochemistry assay indicates the physiological temperament of animals to their nutrition and health status. Upon supplementation of swine and dairy origin probiotics decreased serum glucose concentration in grower pigs, which was consistent due to period and interaction of treatment x period (Table-3). In the same line, Cui *et al*. [27] also observed lower blood glucose concentration by supplementing *Bacillus* *subtilis* in crossbred pigs. A positive effect was observed on serum globulin and A: G ratio without showing any significant effect on serum protein and albumin due to the supplementation of *P. acidilactici* FT28 in growing crossbred pigs (Table-3). The positive effect of *P. acidilactici* FT28 was consistent with decreased concentration of serum triglycerides. In a previous study, Dowarah *et al*. [8] also observed lower serum triglyceride level by supplementing species-specific *P. acidilactici* and *L. acidophilus* in grower-finisher pigs. Du Toit *et al*. [28] observed bile salt hydrolase activity of gut-associated LAB, which may be responsible for de-conjugation of bile salts and results in decreased blood cholesterol. Therefore, this may also account lower serum concentration of cholesterol in *P. acidilactici* FT28 (fecal origin)-fed animals in comparison to control and *L. acidophilus*. Similar to our result, Dhruw *et al*. [24] observed lower total blood cholesterol level by supplementing *L. acidophilus* NCDC15 and curd in weaning piglets, which was also confirmed by incorporation of probiotic in broilers [29,30].

Probiotic-supplemented groups showed higher carcass weight, dressing percentage, and vital organ weight as compared to control (Table-4). Similarly, Kumar *et al*. [31] reported that supplementation of probiotic at 5 g/pig/day increased carcass weight, dressing percentage, and meat percentage in pigs. In contrast, Anna *et al*. [32] and Dowarah *et al*. [17] did not observe any significant effect on carcass weight and dressing percentage by supplementing probiotics in grower-finisher pigs. In the present study, no significant effect was observed on loin muscle pH, WHC, and ERV in probiotic-supplemented groups compared to control (Table-4). In contrast, previous studies reported higher pH and WHC value in meat with dietary supplementation of probiotics in finishing pigs [33-35]. The CP and total ash content of loin muscle were higher in *P. acidilactici* FT28 group compared to *L. acidophilus* NCDC15, which was higher than control animals. The high CP content in loin muscle might be due to higher nitrogen retention and CP digestibility in the supplemented groups, though opposite results were observed from the previous studies by supplementing different sources of probiotics in grower-finisher pigs [36,37]. Earlier studies also showed that supplementation of *Lactobacilli* spp. reduced intramuscular fat deposition in growing-finishing pigs by the inhibition of lipoprotein lipase activity [9]. Lower EE content of loin meat in both probiotic-supplemented groups was confirmed in our study. Similarly, Sevarolli [38] also reported lower EE content of loin meat by the dietary treatment of *L. acidophilus* in grower-finisher pigs.

The epithelial lining of the small intestine has finger-like projections known as villi, which help to increase its surface area for digestion and absorption processes [39]. In addition, the mucosal surface of the small intestine has a tubular gland that opens into the lumen at the base of the villi known as crypts. For optimal function of the small intestine, long villi are desirable. The villi length and crypt depth were significantly improved in both probiotic-supplemented groups (Table-4 and Figure-1). Increased villi height and crypt depth were supported with increased nutrient digestibility and retention of nitrogen in supplemented animals. Previous study also showed that, of *P. acidilactici* and *L. acidophilus*, increased height, crypt depth, and V:C ratio in grower-finisher pigs [8].

## Conclusion

From the findings of the present experiment, it may be suggested that supplementation of probiotic of dairy origin (*L. acidophilus* NCDC 15) and swine origin (*P. acidilactici* FT28) is beneficial in growing pigs in respect of growth, feed conversion efficiency, and digestibility of nutrients. The supplementation also improved blood biochemical profile, meat quality, and intestinal morphology in growing pigs. Hence, it may be concluded that probiotics of dairy and swine origin may be fed for better production in growing pigs at 200 g per day per pigs. Moreover, the probiotic of swine origin is more beneficial over the probiotic of dairy origin in respect to nitrogen retention, blood globulin, and lipid profile.

## Authors’ Contributions

MJ was responsible for conducting animal trial and laboratory analysis; BNS and RD planned experimental design, statistical analysis, and writing of the manuscript; ST was involved in blood biochemical analysis; DK was responsible for the supply of experimental animals and feeds. KBDC involved in histopathological observation and measurement. All authors read and approved the final manuscript.
